# 转化性小细胞肺癌研究进展

**DOI:** 10.3779/j.issn.1009-3419.2021.101.28

**Published:** 2021-10-20

**Authors:** 爽 张, 双 李, 雅楠 崔, 颖 程

**Affiliations:** 130000 长春，吉林省肿瘤医院胸部肿瘤内科 Department of Thoracic Oncology, Jilin Cancer Hospital, Changchun 130000, China

**Keywords:** 肺肿瘤, 转化, 机制, 治疗, Lung neoplasms, Transformation, Mechanism, Therapy

## Abstract

小细胞肺癌（small cell lung cancer, SCLC）转化是非小细胞肺癌尤其是表皮生长因子受体（epidermal growth factor receptor, *EGFR*）突变患者的重要耐药机制之一。目前发现转化性SCLC具有与原发性SCLC相似的临床特征，对化疗短期有效，中位生存期仅1年左右。*RB1*缺失、体细胞拷贝数改变与SCLC转化有关，但发生SCLC转化的确切分子机制仍不完全清楚。转化性SCLC的治疗也面临巨大的挑战，针对SCLC的化疗方案是目前的主要治疗选择，联合治疗、局部治疗以及预防SCLC转化的策略也在探索。本文将转化性SCLC的临床特征、分子机制和治疗选择进行综述。

## Abstract (en)

The transformation of non-small cell lung cancer to small cell lung cancer (SCLC) is one of the major resistant mechanisms, especially patients with epidermal growth factor receptor mutant lung adenocarcinoma. Translational SCLC has been found to have similar clinical features to primary SCLC. Chemotherapy was short-term effective for transformational SCLC, with a median survival of only about 1 year. The deletion of *RB1* and the change of somatic copy number were associated with SCLC transformation. Although the molecular mechanism of SCLC transformation is still not fully understood. At the same time, the treatment of transformational SCLC also faces great challenges. Currently, chemotherapy regimens for SCLC are the main treatment options for transforming SCLC. Combination therapy, local treatment and strategies for prevention of SCLC transformatio are also being explored. This article will review research advances on the clinical features, molecular mechanism and treatment options of translational SCLC.

小细胞肺癌（small cell lung cancer, SCLC）转化是表皮生长因子受体（epidermal growth factor receptor, *EGFR*）突变非小细胞肺癌（non-small cell lung cancer, NSCLC）患者经酪氨酸激酶抑制剂（tyrosine kinase inhibitor, TKI）治疗后的耐药机制之一。随着再活检的增加，发现SCLC转化并不限于特定分子亚型的NSCLC，也不限于特定的治疗。为了与原发性SCLC区别，将其称为转化性SCLC。转化性SCLC是肿瘤进化的结果，但确切的分子机制仍然在研究中。转化性SCLC与原发性SCLC的临床特征相似，针对原发性SCLC的化疗方案是转化性SCLC最常见的治疗选择，更加有效的治疗策略还在探索中。

## 1 抗肿瘤治疗后转化性SCLC的临床特点

2006年纪念斯隆-凯特琳癌症中心^[1]^报道了1例45岁不吸烟的女性肺癌腺患者接受EGFR-TKI治疗和化疗后疾病进展，再次活检病理显示为突触素阳性的SCLC，而且基因分析发现存在*EGFR*外显子19缺失突变，从此SCLC转化逐渐被认识。研究^[2]^发现5%-15%的*EGFR*突变的NSCLC疾病演进过程中会经历SCLC转化。虽然SCLC转化更常见于*EGFR*突变NSCLC经TKI治疗进展的患者，但并不限于*EGFR*突变的患者，间变性淋巴瘤激酶（anaplastic lymphoma kinase, *ALK*）融合突变^[3]^、*ROS1*突变的腺癌^[4]^，*EGFR*野生型的腺癌^[5]^以及肺鳞癌接受免疫治疗的患者^[6]^也有发生SCLC转化的报道，可见抗肿瘤治疗后发生SCLC转化是一种重要的临床现象。因此临床上将转化性SCLC作为一种特殊类型的肺癌。

*EGFR*突变NSCLC患者TKI治疗后发生SCLC转化的中位时间在13个月-18个月^[7-9]^。多数转化性SCLC与原NSCLC分享相同的*EGFR*突变，但是与原NSCLC相比，EGFR蛋白表达减少，对原来的TKI治疗不再敏感^[10]^。转化性SCLC的生物学行为与原发性SCLC相似，基因组分析发现转化性SCLC也普通存在*TP53*和*RB1*缺失^[8]^，更具侵袭性，疾病迅速进展。与原发性SCLC相比，转化性SCLC患者年龄更轻，不吸烟、轻度吸烟的患者比例高，男性和女性发生比例相似^[8]^。目前，转化性SCLC缺少标准治疗选择。针对原发性SCLC的化疗方案是最常用的治疗选择，通常短期有效，转化后中位的无进展生存时间（progression-free survival, PFS）在3.5个月左右^[4, 7, 8]^，转化后中位生存时间（overall survival, OS）约10个月^[8, 9]^。一代、二代TKI与三代TKI治疗后发生SCLC转化后的PFS和OS没有显著差异^[9]^。

## 2 转化性SCLC分子机制研究进展

### 2.1 转化性SCLC发生的假说

目前关于转化性SCLC的发生有几种假说。①诊断前存在NSCLC和SCLC两种组织学成分：尽管Tenjin等^[11]^的研究对诊断时的肿瘤组织进行了仔细地检查，没有发现SCLC成分，但有研究者^[12]^认为最初NSCLC诊断的组织标本有限，不能完全排除有非常低比例的SCLC存在，现有的检查手段和方法难以发现；然而如果患者在诊断时就混有SCLC成分，TKI治疗有效地抑制了*EGFR*突变NSCLC成分，而对TKI不敏感的SCLC成分具有快速增殖的特点，短期将出现疾病进展，这与发生SCLC转化前患者有相对较长的缓解期，转化为SCLC后才爆发性进展不符^[10]^；②转化性SCLC和转换前的NSCLC是共同的前体细胞分支进化的结果^[13]^。Lee等^[13]^对4例SCLC转化前后配对的肿瘤组织进行了全基因组检测和克隆相关性分析，研究发现转化性SCLC克隆并不直接来源于肺腺癌的主克隆，而且尽管SCLC转化是肿瘤进化的晚期事件，但克隆分支可能出现在TKI治疗之前，甚至是诊断肺癌之前。Xie等^[14]^也认为转化性SCLC并不是由肺腺癌直接进化而来，而是在诊断前经过分支进化而来。对于NSCLC何时以何种方式发生SCLC转化，具体的机制仍不完全清楚；③转化性SCLC是一种第二原发肿瘤^[5]^：然而多项研究^[15, 16]^发现转化性SCLC中保持了原NSCLC的*EGFR*突变，而原发性SCLC中*EGFR*突变非常罕见，提示转化性SCLC并不是独立于原NSCLC的第二原发肿瘤。

### 2.2 基因改变与SCLC转化

研究者^[9]^发现转化前的NSCLC中*RB1*失活是普遍现象。研究^[10]^也发现*RB1*和*TP53*完全失活富集在发生SCLC转化的患者，*RB1*和*TP53*完全失活的*EGFR*突变肺腺癌发生SCLC转化风险是无*RB1*和*TP53*失活患者的43倍。Offin等^[17]^的研究进一步证实：*EGFR*/*RB1*/*TP53*三突变的NSCLC有独特的发生SCLC转化的风险。提示肺腺癌中存在*RB1*和*P53*失活是发生SCLC转化的先决条件，是SCLC转化的强有力的预测标志物^[13]^。但仅*RB1*失活并不足以诱导肺腺癌向SCLC转化^[10]^，研究发现Notch信号通路活化^[11]^、*MYC*扩增、*PIK3CA*突变^[14]^和载脂蛋白B mRNA编辑酶催化多肽（apolipoprotein B mRNA-editing catalytic polypeptide, APOBEC）超突变^[13, 17]^也参与SCLC转化^[14]^。

### 2.3 体细胞拷贝数改变与SCLC转化

除了基因改变外，体细胞拷贝数的改变也与SCLC转化相关。研究者^[14]^分析了5例SCLC转化前后配对肿瘤组织标本的体细胞拷贝数改变（copy number variations, CNV），发现SCLC转化后CNV负荷显著增加，而且CNV负荷越高的肺腺癌，发生SCLC转化的时间越短，转化性SCLC的CNV负荷越高，转化后的生存时间也越短（39.0个月*vs* 61.1个月）。提示CNV促进了SCLC转化的发生。CNV负荷与发生SCLC转化的时间和转化后的生存相关，但高CNV负荷能否预测SCLC转化还需要进一步研究。

## 3 转化性SCLC治疗进展

化疗是目前转化性SCLC最常见的治疗选择。转化性SCLC虽然常常存在原有的*EGFR*突变，但EGFR蛋白表达减少，对TKI治疗耐药^[10]^，因此SCLC转化后TKI常与化疗联合使用或者作为化疗后的维持治疗^[5, 8]^。选择的TKI既有沿用SCLC转化前应用的TKI，也有更换另外的TKI进行治疗的，但尚缺少关于沿用原TKI治疗与更换其他TKI治疗对转化性SCLC疗效的研究。目前转化性SCLC的治疗包括针对发生SCLC转化后的治疗以及防止、延缓NSCLC向SCLC转化的策略。针对转化后SCLC的治疗缺少前瞻性研究结果，主要依据为小样本的回顾性研究和病例报告的数据。预防和延缓SCLC转化的策略主要依据转化性SCLC机制探索中发现的一些分子改变，以及细胞系和动物模型的结果。

### 3.1 针对转化性SCLC的治疗

#### 3.1.1 标准化疗

化疗是转化性SCLC首要的治疗选择。一项转化性SCLC的多中心回顾性研究^[9]^中，SCLC转化后最常选择的治疗方案是依托泊苷/顺铂（Etoposide/Platinum, EP）方案，客观缓解率（overall response rate, ORR）为44.4%（12/27），疾病控制率（disease control rate, DCR）达74.1%，中位PFS是3.5个月。另一项回顾性研究^[5]^纳入了61例*EGFR*突变型和*EGFR*野生型NSCLC治疗后发生SCLC转化的患者，这项研究中EP方案依旧是SCLC转化后主要的治疗选择。*EGFR*突变型和*EGFR*野生型患者发生SCLC转化后对EP方案的应答相似（ORR分别为45%和40%）。除了EP方案外，伊立替康/铂类（Irinotecan/Platinum, IP）治疗也是转化性SCLC的一种治疗选择^[9]^，3例患者中2例患者应答，DCR为100%。也有研究者^[8]^在SCLC转化后尝试了紫杉醇或者含紫杉醇方案的治疗，ORR为50%，PFS为2.7个月。从这些研究中看到转化性SCLC对化疗的ORR略低于原发性SCLC一线治疗，但高于复发SCLC患者，中位PFS与复发SCLC相似。

#### 3.1.2 联合治疗

仅有40%-50%的转化性SCLC对单纯的化疗有应答，而且缓解的时间短暂。在化疗的基础上联合其他药物的研究也在尝试。Fujita等^[18]^报告了1例*ALK*融合突变SCLC转化的患者接受伊立替康联合阿来替尼治疗获得了部分缓解（partial response, PR）^[3]^。此外，Lai等^[19]^也报告了2例*EGFR*突变NSCLC经TKI治疗后发生转化SCLC的患者分别采用单纯化疗和化疗联合TKI治疗，仅EP方案化疗的患者获得3个月的PFS，接受了EP联合TKI治疗的患者，获得了8个月的PFS。提示*EGFR*突变NSCLC发生SCLC转化的患者采用SCLC标准化疗方案联合TKI治疗是值得尝试的。一项小样本回顾性研究^[20]^比较了单纯化疗（EP/IP方案）与联合方案（EP/IP方案+贝伐珠单抗或者TKI）在*EGFR*突变NSCLC经TKI治疗后发生SCLC转化患者的疗效，研究纳入21例患者，单纯化疗组12例，联合治疗组9例，研究发现联合治疗的ORR和PFS显著优于化疗（ORR：50% *vs* 25%，*P*=0.002；PFS：6.4个月*vs* 2.9个月，*P*=0.024），联合治疗的OS在数值上更长（10.7个月*vs* 7.1个月，*P*=0.237），有获益趋势，研究提示联合治疗可能是转化性SCLC患者更有效的治疗策略，是值得开展前瞻性研究的重要方向。这项研究目前是在世界肺癌大会进行了报道，尚未正式发表，也缺少关于是否患者沿用原TKI治疗还是更换了其他的TKI的信息，TKI药物种类的选手是否影响患者的PFS和OS也需要进一步的研究。

除了在化疗的基础上联合TKI或者贝伐珠单抗的治疗外，TKI基础上联合其他治疗也进行了探索。磷酸激酶分析发现转化性SCLC细胞系中AKT/mTOR信号途径显著活化，转化性SCLC细胞系对AKT抑制剂或者TKI联合AKT抑制剂都非常敏感^[21]^。提示PI3K/AKT/mTOR抑制剂联合TKI可能是转化性SCLC治疗的又一研究方向。此外研究^[21]^发现转化性SCLC细胞系中Sin3和NuRD显著上调，Sin3和NuRD是组蛋白脱乙酰化酶（histone deacetylase, HDAC）的重要复合物，通过HDAC介导转录调节。研究发现与*EGFR*突变NSCLC细胞系相比，转化性SCLC细胞系对HDAC抑制剂更敏感，提示HDAC抑制剂联合TKI可能也是转化性SCLC治疗的探索方向。

#### 3.1.3 分子靶向单药治疗

一项回顾性研究^[9]^中有5例转化性SCLC患者接受安罗替尼治疗，获得66.7%的ORR，中位PFS为6.2个月。安罗替尼在复发的NSCLC和复发SCLC的治疗中都获得较好的疗效，可能也是转化性SCLC值得尝试的治疗策略。细胞系的研究发现BCL-2抑制剂对部分SCLC敏感，BCL-2抑制剂是SCLC患者潜在的靶向治疗药物。Niederst等^[10]^的研究发现转化性SCLC的细胞系对BCL-2抑制剂更敏感，但BCL-2抑制剂能否成为转化性SCLC的治疗选择还需要进一步研究明确。

#### 3.1.4 免疫治疗

免疫治疗已经成为缺少驱动突变NSCLC的重要治疗选择，而多数存在驱动突变的患者对免疫治疗反应欠佳。研究^[22]^也发现*EGFR*突变NSCLC常呈现为免疫抑制表型：程序性细胞死亡受体配体1（programmed cell death ligand 1, PD-L1）低表达，低肿瘤突变负荷，细胞毒性T细胞减少，高表达CD73，LAG-3上调^[23]^。多数SCLC也是免疫抑制表型，缺少主要组织相容性复合物-I（major histocompatibility complex-I, MHC-I）表达^[24]^，转化性SCLC的MHC-I表达降低^[26]^，一项回顾性研究^[8]^中报告了17例转化性SCLC接受免疫检查点药物治疗并没有患者出现应答，这些研究提示SCLC转化前后都不是免疫治疗的理想类型。这可能与转化性SCLC存在显著的抗原提呈功能缺陷有关。目前关于转化性SCLC免疫微环境特征的研究非常有限，而且关于转化性SCLC免疫治疗也仅为个案报道，免疫治疗在转化性SCLC中的治疗价值还需要更多的研究。

#### 3.1.5 局部治疗

*EGFR*突变NSCLC按照疾病进展模式分为局部进展、缓慢进展和快速进展。转化性SCLC的患者也有呈局部进展的。Pignataro等^[25]^报告1例以寡转移灶局部进展的转化性SCLC，患者奥西替尼治疗后仅出现肺部新寡转移灶，经病理证实为SCLC，患者停止奥西替尼治疗，接受EP方案治疗3个周期及胸部病灶放疗（60 Gy/30 f）后胸部新病灶获得PR，但脑部病灶进展，患者重新开始了奥西替尼治疗，8个月后脑部病灶也获得完全应答，胸部病灶未进展。这项病例报告提示转化性SCLC的病灶存在异质性，而且是动态演变的，不同的时间需要采取不同的策略。

### 3.2 预防、延缓SCLC转化的策略

转化性SCLC更具侵袭性，治疗更困难。如果对高风险发生SCLC转化的患者给予适当的治疗，延缓或预防SCLC转化的发生，可能有助于改善患者的预后。

#### 3.2.1 化疗预防策略

Lee等^[13]^发现具有*RB1*和*TP53*失活的NSCLC患者发生SCLC转化的风险显著增加。Offin等^[17]^进一步证实*EGFR*/*TP53*/*RB1*三突变NSCLC富集了发生SCLC转化的人群，而且相对于*EGFR*/*TP53*双突变和仅存在*EGFR*突变的患者，*EGFR*/*TP53*/*RB1*三突变NSCLC患者TKI治疗的PFS更短（*EGFR*/*TP53*/*RB1*三突变*vs* *EGFR*/*TP53*双突变*vs* *EGFR*：9.5个月*vs* 12.3个月*vs* 36.6个月，*P*=2×10^-9^）。对*EGFR*/*TP53*/*RB1*三突变NSCLC患者，如果TKI治疗的同时给予EP方案化疗，理论上既可以抑制大部分对TKI治疗敏感的克隆，还能针对潜在发生SCLC转化的克隆，可能是延缓*EGFR*/*TP53*/*RB1*三突变NSCLC疾病进展、预防SCLC转化的策略。目前一项奥西替尼联合EP方案治疗*EGFR*/*TP53*/*RB1*三突变NSCLC的前瞻性I期研究（NCT03567642）正在进行。

#### 3.2.2 靶向治疗预防策略

转化性SCLC普遍存在*RB1*缺失。RB1阻止G_1_期-S期细胞周期检查点的通过，发挥细胞周期负性调控因子的作用，*RB1*异常增加复制应激，可能从针对DNA损伤修复的药物中获益^[27]^。*TP53*/*RB1*缺失的前列腺癌中也会出现神经内分泌转化，研究^[28]^发现*TP53*/*RB1*缺失的前列腺癌细胞对聚腺苷二磷酸核糖多聚酶（poly ADP-ribose polymerase, PARP）抑制剂联合ATR抑制剂产生显著的应答。提示TKI联合靶向DNA损伤修复检查点药物的治疗模式可能是防止*EGFR*/*TP53*/*RB1*三突变NSCLC发生SCLC转化的策略。另外药物筛选的研究^[29]^中，发现*RB1*突变的细胞系对Aurora激酶抑制剂非常敏感，而三代TKI治疗*RB1*/*EGFR*突变NSCLC细胞系会诱导极光激酶A（aurora kinase A, AURKA）活化^[30]^，活化的AURKA将抑制三代TKI导致的凋亡并诱导耐药，因此Aurora激酶抑制联合TKI可能也是充满前景的预防SCLC转化的策略。目前有一项Aurora激酶抑制联合奥西替尼治疗*EGFR*突变NSCLC的I期/Ib期研究正在进行（NCT04085315）。

#### 3.2.3 表观遗传学的预防策略

EZH2是多梳抑制性复合物2（polycomb repressive complex 2, PRC2）的催化成分，PRC2活性增加能促进和维持细胞的可塑性^[31]^，在前列腺癌祖系可塑性研究^[32]^发现，发生小细胞转化的前列腺癌中EZH2高表达，并且表达特殊DNA甲基化谱，应用EZH2抑制剂能够抑制前列腺癌神经内分泌转化。在*RB1*缺陷的*EGFR*突变NSCLC也存在*EZH2*异常^[33]^，TKI联合EZH2抑制剂也可能是阻止发生SCLC转化的策略。

## 4 总结

转化性SCLC是肿瘤对抗治疗、进化适应的一种途径。虽然目前研究认为*RB1*/*TP53*失活、*APOBEC*超突变以及CNV改变会促进NSCLC克隆进化，在发生SCLC转化中发挥了重要作用，但是对于足以诱导SCLC转化的关键因子仍在探索中。单细胞测序是研究肿瘤异质性和克隆演化的重要方法，未来通过单细胞测序可能有助于明确转化性SCLC的起源，发现驱动SCLC转化的关键分子事件。另外SCLC根据转录因子的差异分为不同的亚型，原发性SCLC的分子分型是否也适用于转化性SCLC，肿瘤周围的基质细胞、免疫细胞以及异常的肿瘤血管，缺氧的肿瘤微环境在SCLC转化的过程中发挥怎样的作用，肿瘤微环境对转化性SCLC后续治疗的影响都是未来探索的方向。
